# Supplementary material for: Identification of photoperiod-induced specific miRNAs in the adrenal glands of Sunite sheep (Ovis aries)
Source: Front Vet Sci. 2022 Jul 22;9:888207. doi: 10.3389/fvets.2022.888207 (PMC9354845; doi:10.3389/fvets.2022.888207)
Supplement: Supplementary file 1 [file Data_Sheet_1.ZIP › Supplementary file/miRDeep2 file.zip!/miRDeep2 file/Know_Structure/LP42A2/oar-mir-194.pdf]

miRBase precursor : oar-mir-194
Total read count : 5012
oar-miR-194 read count : 5008
remaining reads : 4

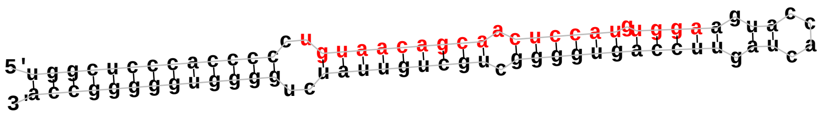

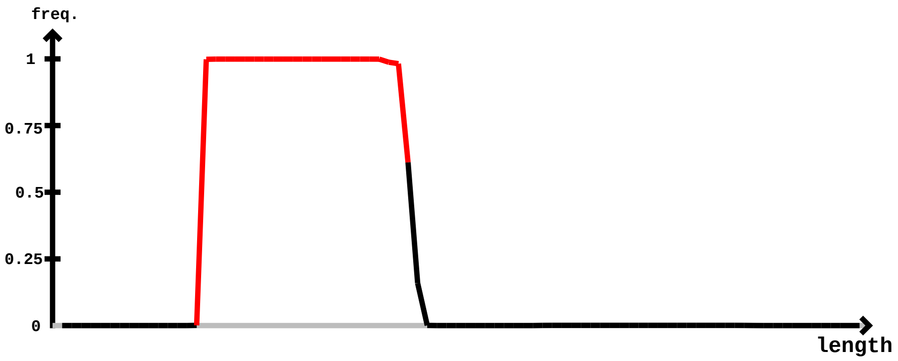


```
                 oar-miR-194
5'- uggcucccacccccuguaacagcaacuccaugugaaguaccacuaguuccaguggggcugcuguuaucuggggugggggcca   -3'    exp
    ((((((((((((..(((((((((.((((((.(((((.((....)).)))))))))))).)))))))))..))))))))))))    reads      mm         sample
    .............cuguaacagcaacuccaugugg................................................    1          0          seq
    .............cuguaacagcaacuccaugugga...............................................    2          0          seq
    .............cuguaacagcaacuccauguggaa..............................................    1          0          seq
    ..............uguaacagcaacuccaug...................................................    1          0          seq
    ..............uguaacagcaacuccaugu..................................................    58         0          seq
    ..............uguaacagcaacuccaugug.................................................    27         0          seq
    ..............uguaacagcaacuccaugugg................................................    1852       0          seq
    ..............uguaacagcaacuccaugugga...............................................    2266       0          seq
    ..............uguaacagcaacuccauguggaa..............................................    777        0          seq
    ..............uguaacagcaacuccauguggaC..............................................    16         1          seq
    ..............uguaacagcaacuccauguggaag.............................................    3          0          seq
    ...............guaacagcaacuccaugugg................................................    2          0          seq
    ...............guaacagcaacuccaugugga...............................................    1          0          seq
    ...............guaacagcaacuccauguggaa..............................................    1          0          seq
    .................................................ccaguggggcugcuguuaucu.............    1          0          seq
    .................................................ccaguggggcugcuguuaucug............    2          0          seq
    ..................................................caguggggcugcuguuaucugg...........    1          0          seq
```
